# Dynamic–Static Perceptual Weight Reversal in 3D-Printed Climbing Holds: A Multi-Modal Biomechanical and Sensory Study

**DOI:** 10.3390/s26216863

**Published:** 2026-10-30

**Authors:** Di Niu, Hanyi Wang, Pengcheng Gao, Junjie Wang

**Affiliations:** 1 School of Physical Education, Huazhong University of Science and Technology, Wuhan 430074, China; ddd_niu@hust.edu.cn; 2 Faculty of Materials Science and Chemistry, China University of Geosciences, Wuhan 430074, China; 1202511563@cug.edu.cn; 3 School of Automobile and Traffic Engineering, Wuhan University of Science and Technology, Wuhan 430081, China; 4 School of Physical Education, China University of Geosciences, Wuhan 430074, China

**Keywords:** climbing holds, 3D printing, sensory perception, biomechanics, perceptual weight reversal

## Abstract

**Highlights:**

**What are the main findings?**

- A dynamic–static perceptual weight reversal was identified: surface pattern dominates perception during dynamic climbing, while material stiffness dominates during static grasping.
- A three-layer association model (physical → biomechanical → sensory) reveals progressively decreasing correlation strength across layers, with biomechanical metrics as the critical intermediary.

**What are the implications of the main findings?**

- Hold design should be context-specific: dynamic-movement holds prioritize surface texture, while static-resting holds prioritize material stiffness tuning.
- The 22.7-fold inter-individual variation in grip force supports data-driven, 3D-printed hold customization tailored to individual biomechanical and perceptual profiles.

**Abstract:**

This study investigated how material stiffness and surface texture differentially influence climbers’ sensory perception and biomechanical force patterns under dynamic versus static conditions. Twelve climbing hold designs—three materials (PLA, PEBA, TPU) crossed with four surface patterns—were tested on 30 climbers across three skill levels using sensory evaluation, six-axis force measurement, and physical characterization. Surface pattern dominated perception during dynamic climbing, while material properties became primary during static grasping, constituting a dynamic–static perceptual weight reversal. A three-layer association model revealed progressively decreasing correlation strength from physical properties through biomechanical force to sensory perception. Machine learning identified two grip strategy types varying with skill level. Individual grip force varied 22.7-fold, exceeding material or pattern effects. Gender differences in pattern preference were also observed. These findings challenge the assumption of a single optimal hold design and support context-specific, individualized climbing hold engineering.

## 1. Introduction

Equipment design critically modulates athletic performance and injury risk across sports. Sport climbing’s recent inclusion in the Olympic program has accelerated global participation, intensifying demand for evidence-based equipment design [1,2]. Comprehensive injury reviews further underscore the importance of optimized equipment for climber safety [3]. Among climbing equipment, holds are the critical interface between athlete and wall—their material and surface properties directly influence force production, movement fluency, and perceptual-motor exploration [4,5,6].

Recent advances in additive manufacturing have reshaped hold design possibilities. Three-dimensional printing enables precise fabrication of complex surface textures, rapid design iteration, and cost-effective small-batch customization [7,8]. A systematic review confirmed that 3D printing has improved performance across multiple sports through customized equipment and optimized geometries [9]. Yet the scientific understanding of how different hold properties affect climbers has not kept pace with these technological advances [10].

Three interrelated gaps limit current knowledge. First, research is siloed across analytical layers: most studies address physical friction and biomechanical principles in isolation [11,12], few examine detailed force patterns with instrumented holds [13], and very few integrate physical properties, biomechanical responses, and subjective sensory evaluation into a unified framework. A pioneering study showed that climbers judge holds by perceived grippiness rather than roughness, with grippiness—but not roughness—correlating with friction coefficients [14]; tribological work on climbing shoe rubber further demonstrated the importance of surface interactions [15]. Yet the complete pathway from material properties through biomechanical responses to subjective judgment remains poorly understood [16]. Second, nearly all testing occurs under static conditions [17,18], and hangboard training research relies almost exclusively on static hanging [19,20]. Climbing, however, is fundamentally dynamic, and skill differences are most pronounced during dynamic movement sequences [21]. Emerging evidence suggests dynamic and static contexts engage distinct information-processing mechanisms [22,23]; whether the relative importance of material versus texture shifts between states remains unknown. Third, individual differences are largely ignored [24,25]. Sex differences in climbing physiology are well documented [26], and performance-predictor studies have identified multiple factors correlated with climbing ability [27,28]. Equipment, however, is typically treated as a constant rather than a variable interacting with individual characteristics—a notable oversight given that 3D printing’s greatest strength lies in customization [29].

To address these gaps, the present study investigates how material properties and surface patterns differentially affect biomechanical force patterns and sensory perception under dynamic versus static conditions, and how individual characteristics modulate these effects. We tested 12 climbing hold designs—three materials (PLA, PEBA, TPU) crossed with four surface patterns (Flat, Pitcher plant, Snake skin, Lizard skin)—with 30 climbers across three skill levels using a multi-modal approach that integrates sensory evaluation, six-axis force measurement, and physical characterization. We further leveraged machine-learning methods that researchers have increasingly adopted in sports biomechanics for pattern recognition [30,31,32] and grasp synergy analysis [33,34]. First, a dynamic–static perceptual weight reversal is identified and validated: surface pattern dominates perception during dynamic climbing, whereas material properties become primary during static grasping. Second, a three-layer association model (physical → biomechanical → sensory) is described, revealing progressively decreasing correlation strength across layers. Third, distinct grip strategy types are identified, and a data-driven customization framework for 3D-printed climbing holds is proposed. By revealing that perceptual weights are not fixed but dynamically reweighted by movement state, this study resolves the enduring material-versus-texture debate in climbing hold design and establishes a mechanistic foundation for context-aware, individualized sports equipment engineering.

## 2. Materials and Methods

Thirty climbers (19 male, 11 female) were stratified into three skill levels (*n* = 10 per group): novice (V0–V3), intermediate (V4–V7), and advanced (V8+), based on self-reported bouldering grade. Participants were aged 19–32 years, had 0–23 years of climbing experience, and were free of acute upper-limb musculoskeletal pain at testing. Six participants reported a history of upper-limb injury in the past six months but were asymptomatic and cleared for participation. Handedness was assessed via the Edinburgh Handedness Inventory (26 right-hand dominant, 4 left-hand dominant). Exclusion criteria were a history of finger, hand, or wrist surgery, acute musculoskeletal pain at testing, or inability to complete all 12 hold conditions. All participants provided written informed consent.

The sample size (N = 30) was determined by an a priori power analysis conducted using G*Power 3.1. Assuming a medium effect size (f = 0.25) for the primary within-subjects comparison of material × pattern interaction on grip force, with α = 0.05 and 1 − β = 0.80, a minimum of 28 participants was required for a repeated-measures analysis of variance with 12 within-subject conditions. Enrolling 30 participants provided a 7% attrition buffer. This sample size is consistent with prior biomechanical studies of climbing holds, which typically employ 20–35 participants. The repeated-measures design further enhances statistical power by controlling for inter-individual variability. Post-hoc power analysis confirmed that the achieved sample was sufficiently powered (power > 0.85) to detect the medium-to-large interaction effects observed in this study. Participant demographic characteristics are summarized in Table 1 and Table 2.

A 3 × 4 fully crossed factorial design yielded 12 distinct hold types: three materials (PLA, PEBA, TPU 90A) crossed with four surface patterns (Flat, Pitcher plant, Snake skin, Lizard skin). Material stiffness was characterized by Shore hardness and tensile modulus (Table S1, Supplementary Materials). Patterns were bio-inspired, with feature heights of 900 µm, 400 µm, and 300 µm for the pitcher, snake skin, and lizard skin patterns, respectively [35]. All holds were fabricated by fused-deposition modeling using a Bambu Lab X1 Carbon (Bambu Lab, Shenzhen, China). The standardized geometry (footprint 80 × 60 mm; maximum edge depth 25 mm) followed commercial gym mounting interfaces. Detailed FDM 3D printing parameters for all three materials are provided in Supplementary Table S2.

Grip force was recorded using a custom instrumented climbing hold embedding a six-axis force/torque sensor (model 304B, DTS Sensing System, Shenzhen, China) rigidly mounted to a steel backing plate on a vertical wooden wall. Force data were sampled at 10 kHz. Grip force was defined as the resultant magnitude of the three orthogonal force components (*F*_total_ = (*F*_x_^2^ + *F*_y_^2^ + *F*_z_^2^)^1/2^) and, for cross-participant comparison, was normalized to body weight and reported as relative force (N/kg or %BW). This relative force has been reported, whereas individual force values reported in Newtons (0.65–14.78 N) correspond to the absolute resultant force. Each participant completed two grip conditions in randomized order for each of the 12 hold types: (i) static grip—hang from the hold with relaxed posture for 10 s; and (ii) dynamic grip—perform a controlled pull-up from full arm extension to 90° elbow flexion over 1.5 s, repeated five times. Each condition was repeated three times per hold type, with 30 s inter-trial rest and 5 min inter-hold rest. Task order (static vs. dynamic) was counterbalanced across participants, who were randomly assigned to one of two order groups (half performing static trials first and half dynamic first); within each task, hold order was independently randomized for each participant. To evaluate order, practice, and fatigue effects, task order group and trial number were included as fixed effects in the linear mixed-effects models, and the order main effect was not statistically significant (*p* > 0.05). Thirteen biomechanical features were extracted from the force time-series. Full six-axis force/torque sensor specifications, including measurement ranges and calibration details, are provided in Supplementary Table S3.

Immediately after each grip trial, participants rated the hold on six dimensions (roughness, slipperiness, comfort, sharpness, grip security, overall preference) using a 9-point Likert scale. Ratings were collected with the participant seated and blinded to the hold label. Hold order was randomized across participants. The six evaluation dimensions and their standardized verbal anchors are listed in Supplementary Table S4.

For each of the 12 hold types, static friction coefficients were measured using a GYJ-8002 friction coefficient tester, and kinetic (sliding) friction coefficients were measured following ASTM G115: a normal load of 20 N and a constant sliding speed of 10 mm/s were applied against a polished stainless-steel counterface over a total sliding distance of 50 mm, under ambient laboratory conditions (22–24 °C, relative humidity 45–55%), with three replicate tests per 3D-printed specimen. Additional physical properties measured were water contact angle (sessile drop method), surface roughness (Ra, Rz; profilometer), and Shore hardness (ASTM D2240). All measurements were performed in triplicate in Table 3.

Analyses were performed in R version 4.3.2 and Python version 3.11.7. The significance threshold was α = 0.05. Main effects and interactions of Material (3 levels), Pattern (4 levels), and task condition (static vs. dynamic) were tested using linear mixed-effects models (LMMs). LMMs were implemented in R using the lme4 package, with parameters estimated by restricted maximum likelihood (REML); fixed effects included printing material, surface pattern, task condition, the material × pattern interaction, trial sequence group, and trial number, and participant ID was specified as a random intercept. The Satterthwaite approximation (lmerTest package) was used to compute denominator degrees of freedom and *p*-values. Residual diagnostic checks for normality and homoscedasticity were performed, and variance partitioning was calculated using the partR2 package. Skill level and sex were included as between-subjects factors in separate three-way models. Effect sizes are reported as partial eta-squared (η^2^p). Post-hoc comparisons used Tukey HSD with Bonferroni correction. Grip strategies were identified by k-means clustering on 13 standardized biomechanical features (optimal k = 2, determined by the elbow method and the gap statistic). Clustering stability was validated by bootstrapping (1000 resamples), and the final two-cluster solution had a silhouette coefficient of 0.325. Preference ratings were predicted using k-nearest-neighbors regression with 5-fold cross-validation.

## 3. Results

**Figure 1:**
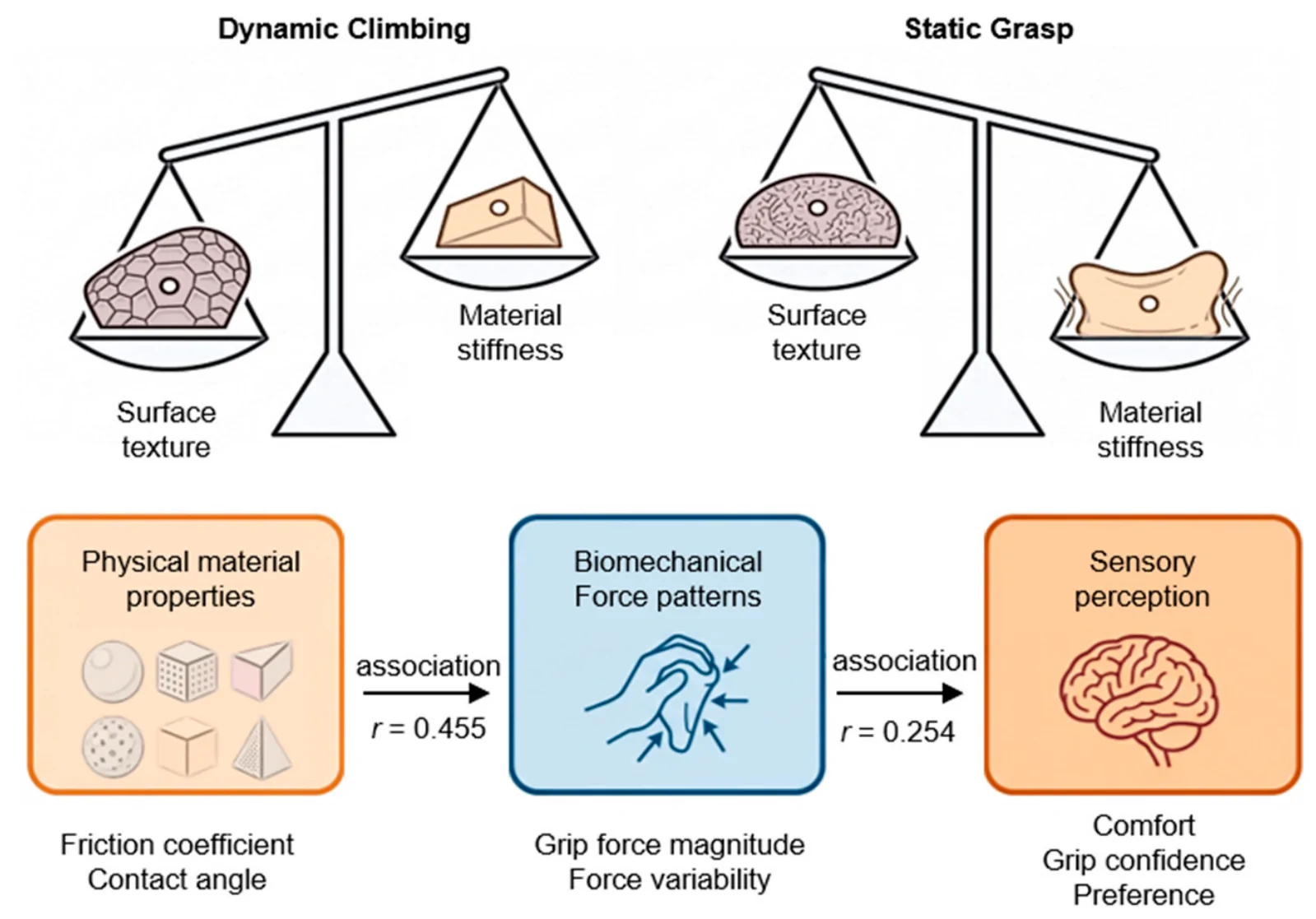
Conceptual framework of the dynamic–static perceptual weight reversal phenomenon and the three-layer association model. (**Left**) Under dynamic climbing conditions, surface pattern exerts a stronger influence on climbers’ sensory perception than material stiffness, as represented by the tilted balance. (**Right**) Under static grasping conditions, the perceptual hierarchy reverses, with material properties becoming the dominant factor. (**Bottom**) The three-layer model illustrates the association chain from physical material properties (friction coefficient, contact angle) through biomechanical force patterns (grip force magnitude, force variability) to subjective sensory perception (comfort, grip confidence, overall preference), with progressively decreasing correlation strength across layers; these cross-sectional correlations describe associations and do not establish causal direction. This integrative framework enables data-driven customization of 3D-printed climbing holds tailored to individual athlete profiles and movement contexts. This schematic was created with the assistance of Nano Banana; the authors reviewed and validated all content for scientific accuracy.

This study addresses a fundamental question in climbing hold design: between material stiffness and surface texture, which factor dominates climbers’ sensory perception, and does this dominance change between dynamic and static movement states? To systematically answer this question, we employed a multi-modal research framework integrating subjective sensory evaluation, objective biomechanical testing, and physical material characterization (Figure 1). A three-layer association model was described, spanning from physical material properties through biomechanical force patterns to sensory perception, with 3D printing technology enabling precise control over material–pattern combinations. This design allowed us to not only quantify perceptual differences but also uncover the underlying mechanistic pathways, ultimately supporting data-driven customization of climbing holds.

### 3.1. Experimental Setup and Instrumented Hold System

**Figure 2:**
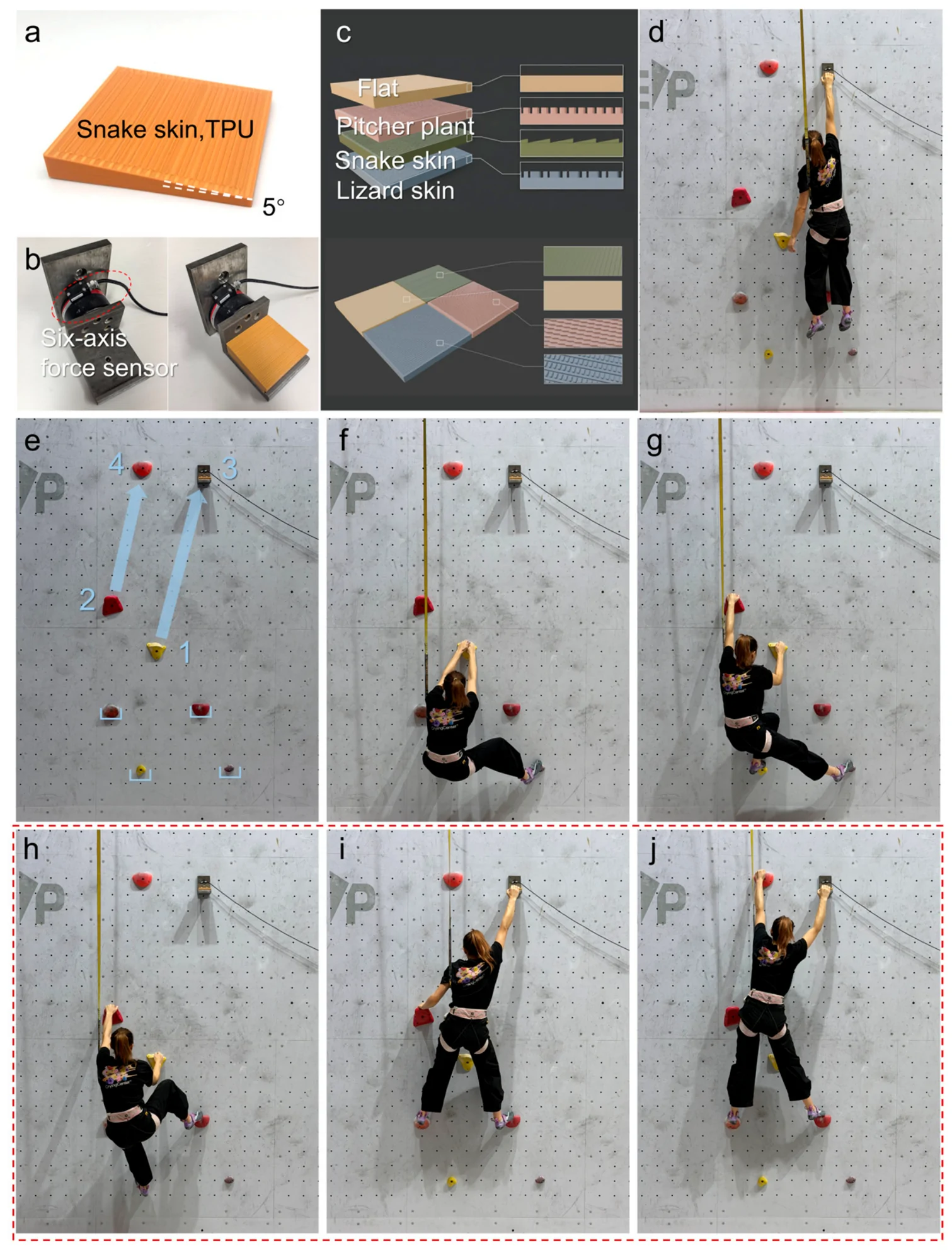
Experimental setup and hold fabrication. (**a**) Close-up photograph of a 3D-printed climbing hold with the snake skin surface pattern in TPU material, showing the parallel micro-ridge texture and the 5° contact-surface inclination angle. (**b**) Six-axis force/torque sensor mounting assembly. Left: sensor fixed to an L-shaped metal bracket without the hold installed. Right: complete assembly with a 3D-printed hold mounted on the sensor; the signal output cable is visible. (**c**) The four material–pattern hold designs used in static force measurement (top row, top-view) and their corresponding surface texture details (bottom row, magnified). (**d**) Static climbing test scenario: a female climber traversing an indoor climbing wall, with the right hand gripping the instrumented hold and the body suspended. (**e**) Bouldering wall test route layout. Handholds 1–4 are labeled, and blue arrows indicate the prescribed movement sequence between handholds. The instrumented hold is mounted at position 3, with the signal cable extending to the right; portions of the instrumented hold surface are visible in the lower right corner. (**f**–**j**) The dynamic movement sequence. The red-dashed bounding box denotes the frame interval corresponding to the dynamic force data recording window.

The experimental setup comprised three coordinated components (Figure 2a–e): 12 3D-printed climbing holds, a custom instrumented hold with a six-axis force/torque sensor, and a standardized bouldering wall protocol.

The holds were printed with precisely controlled material–pattern combinations (3 materials × 4 surface patterns, 12 designs; Figure 2a,c). The custom instrumented hold embedded a six-axis force/torque sensor for simultaneous measurement of Fx, Fy, Fz and Mx, My, Mz at 10 kHz (Figure 2b). The wall protocol prescribed a four-hold movement sequence (Figure 2e). Participants completed both static hanging (10 s; Figure 2d) and dynamic pull-up (5 repetitions; Figure 2f–j) conditions on each of the 12 hold designs, with sensory ratings collected immediately after each trial. The instrumented hold was mounted at position 3 of the movement sequence (Figure 2e), and the red-dashed bounding box in the dynamic sequence (Figure 2f–j) marks the force data recording window.

### 3.2. Dynamic–Static Perceptual Weight Reversal and Gender Divergence

**Figure 3:**
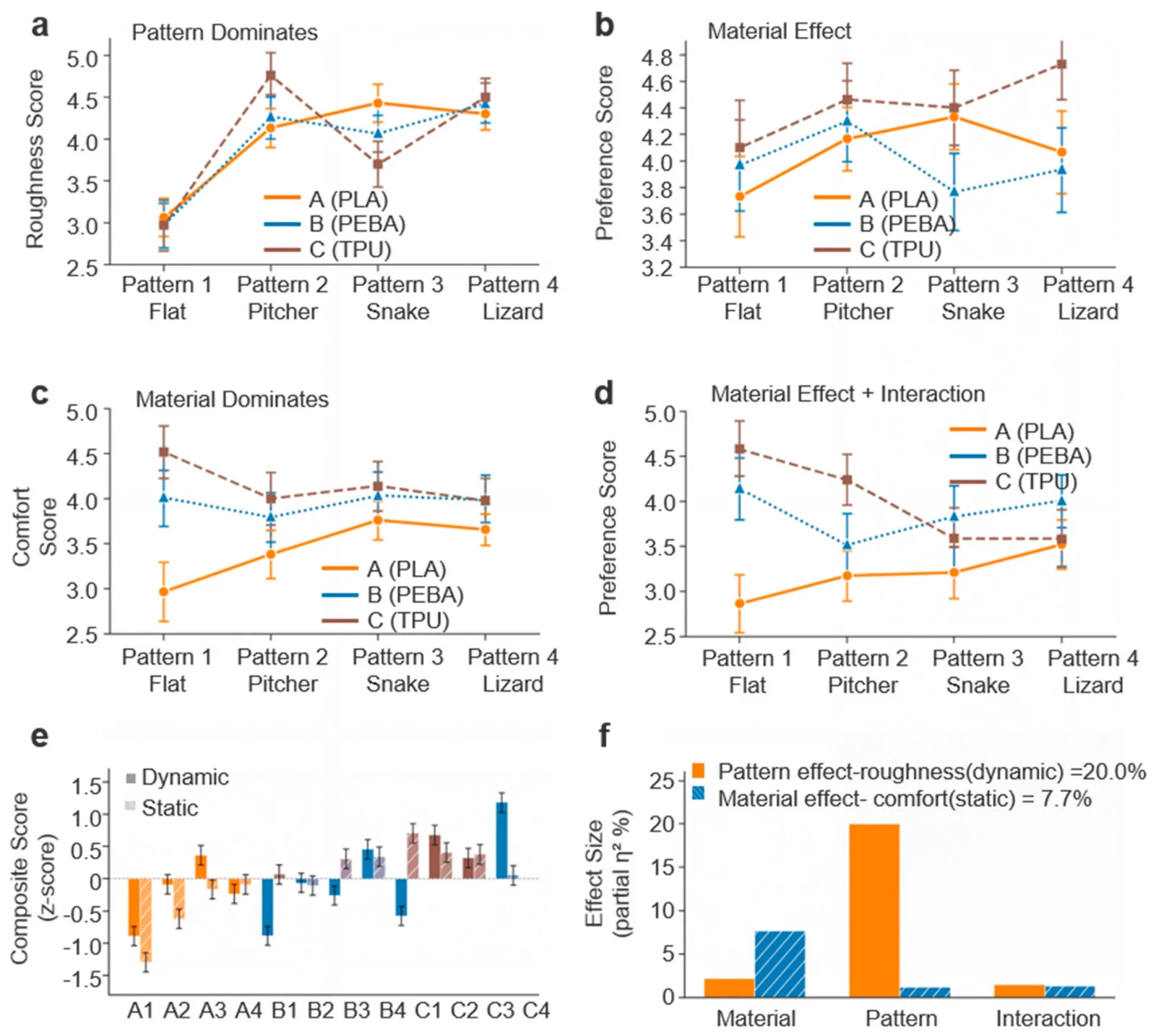
Dynamic–static perceptual weight reversal in sensory evaluation of climbing holds. (**a**) Dynamic roughness perception as a function of surface pattern for three materials (A: PLA, red; B: PEBA, blue; C: TPU, green). (**b**) Dynamic preference ratings across the four surface patterns for each material. (**c**) Static comfort perception across surface patterns and materials. (**d**) Static preference ratings across surface patterns and materials. (**e**) Comprehensive ranking of 12 hold designs under dynamic (left) and static (right) conditions, showing distinct preference hierarchies between movement states. (**f**) Effect size comparison (partial *η*^2^) revealing state-dependent perceptual reweighting: the pattern effect on roughness perception dominates under dynamic conditions (20.0%), whereas the material effect on comfort perception becomes primary under static conditions (7.7%). Data are presented as mean ± standard error. Linear mixed-effects model for dynamic roughness (**a**): material F(2, 53.8) = 2.11, *p* = 0.132, partial *η*^2^ = 0.043; pattern F(3, 81.4) = 6.79, *p* < 0.001, partial *η*^2^ = 0.200; material × pattern F(6, 158.1) = 1.46, p = 0.194, partial *η*^2^ = 0.052. For static comfort (**c**): material F(2, 52.9) = 3.04, p = 0.048, partial *η*^2^ = 0.077; pattern F(3, 77.8) = 1.22, *p* = 0.306, partial *η*^2^ = 0.031; material × pattern F(6, 154.7) = 0.88, *p* = 0.511, partial *η*^2^ = 0.024. Pairwise post-hoc comparisons (Tukey HSD) are reported in Supplementary Table S6.

The most striking finding is a dynamic–static perceptual weight reversal phenomenon. Under dynamic climbing conditions, surface pattern emerged as the dominant factor shaping sensory perception, while material properties took a secondary role. This pattern dominance was most pronounced for roughness perception (Figure 3a), where the four surface textures produced clearly distinct ratings regardless of material type. In contrast, during static grasping, the perceptual hierarchy flipped completely: material stiffness became the primary determinant, and surface texture’s influence diminished significantly across multiple sensory dimensions including comfort and overall preference (Figure 3b,c). The effect size analysis revealed a state-dependent reweighting of perceptual factors: the pattern effect on roughness perception dominated under dynamic conditions (partial *η*^2^ = 20.0%), whereas the material effect on comfort perception became primary under static conditions (partial *η*^2^ = 7.7%) (Figure 3f). Group-level sensory evaluation comparisons across three skill levels and both movement conditions are presented in Supplementary Figure S1; no significant group main effect was observed for any dimension.

**Figure 4:**
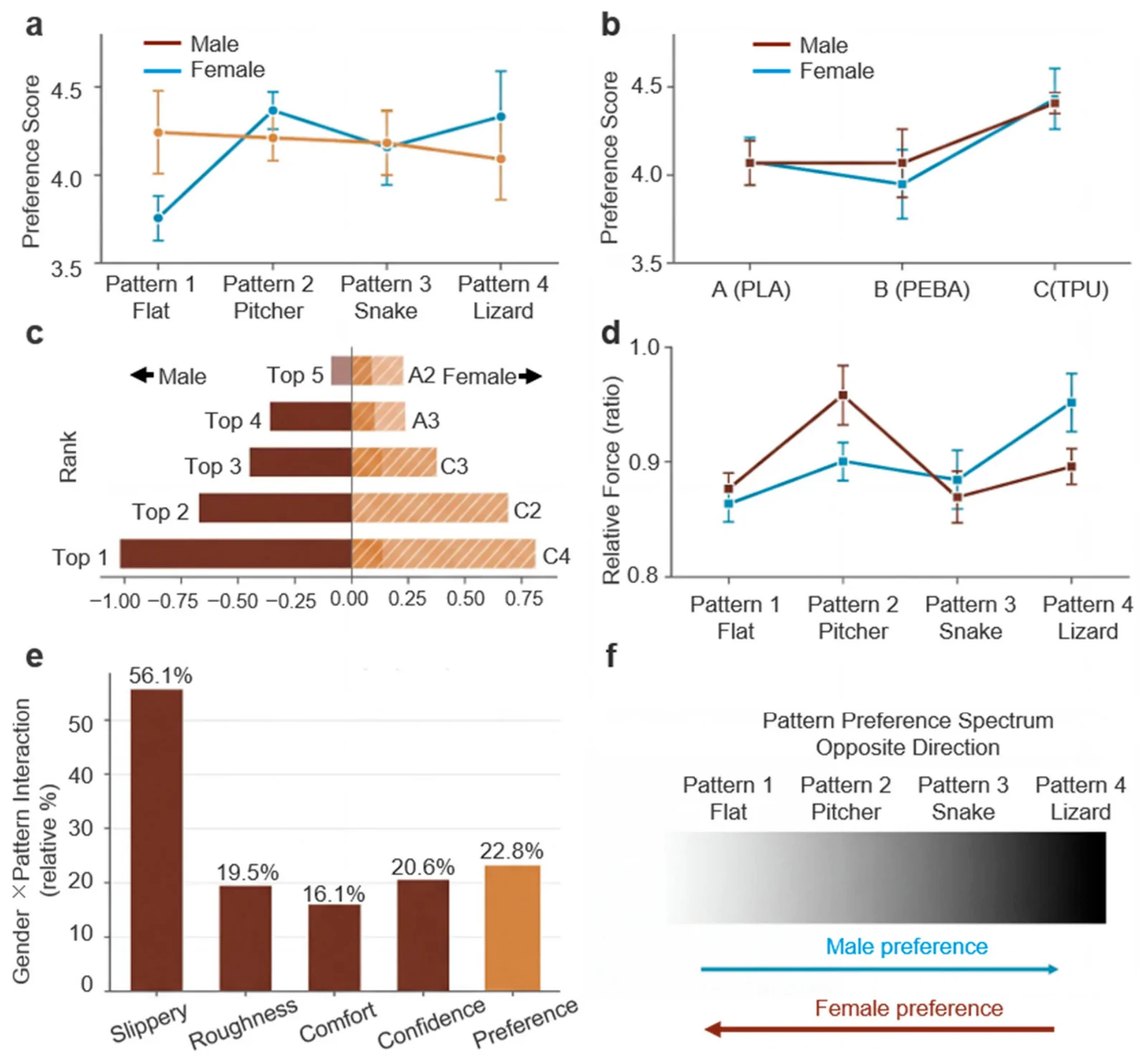
Gender divergence in surface pattern preference and force application. (**a**) Gender × pattern interaction for preference ratings, showing a crossover effect: males favor deeper textures (Pitcher, Lizard) while females prefer shallower surfaces (Flat, Snake). (**b**) Gender × material interaction for preference ratings, with both genders showing identical material preference hierarchies (C > A > B). (**c**) Top 5 hold design rankings for males (left) and females (right), demonstrating minimal overlap between gender-specific preferences. (**d**) Gender × pattern interaction for relative grip force, with males peaking at the Lizard pattern and females at the Pitcher pattern. (**e**) Effect sizes (partial *η*^2^) of gender × pattern interaction across five sensory dimensions, with slippery perception showing the largest interaction effect (56.1%). (**f**) Conceptual illustration of the pattern preference spectrum, highlighting the opposing preference gradients between genders. Data are presented as mean ± standard error.

Beyond the movement state effect, a remarkable gender divergence in surface pattern preference was observed. Male and female climbers showed nearly opposite pattern preference hierarchies: males favored deeper, more aggressive textures (Pitcher and Lizard), while females preferred shallower, smoother surfaces (Flat and Snake) (Figure 4a). This gender difference was specific to pattern preference, not material preference—both genders showed identical material preference hierarchies (C > A > B) (Figure 4b). The gender effect extended to objective force application: males applied maximum force on the Lizard pattern, while females peaked on the Pitcher pattern (Figure 4c). Among all sensory dimensions, slippery perception showed the largest gender × pattern interaction effect (*η*^2^ = 0.561) (Figure 4d). Gender-specific pattern preference scores for the dynamic condition are detailed in Supplementary Table S5, confirming a significant gender × pattern crossover interaction for preference.

### 3.3. Biomechanical Force Patterns and Physical Friction Characterization

**Figure 5:**
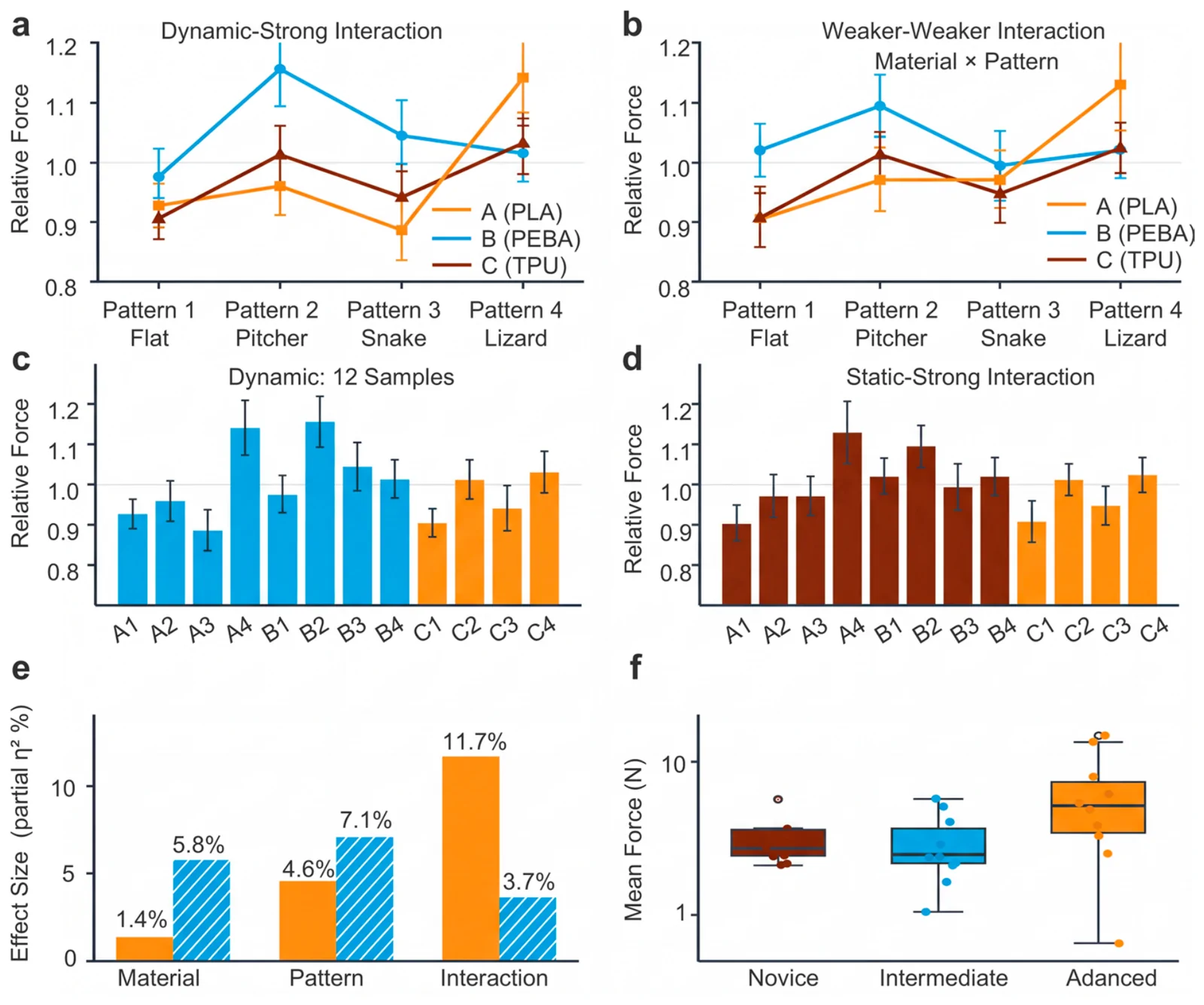
Biomechanical material × pattern interaction under dynamic and static conditions. (**a**) Dynamic grip force as a function of surface pattern for three materials (A: PLA, red; B: PEBA, blue; C: TPU, green), showing strong material–pattern coupling (interaction *η*^2^ = 0.117). (**b**) Static grip force across surface patterns and materials, with substantially weakened interaction effects (*η*^2^ = 0.037). (**c**) Relative grip force values for 12 hold designs under dynamic conditions, grouped by material. (**d**) Relative grip force values for 12 hold designs under static conditions. (**e**) Effect size comparison of material × pattern interaction between dynamic and static conditions, revealing biomechanical parallel to the perceptual weight reversal. (**f**) Individual grip force distribution across three skill levels (novice, intermediate, advanced), demonstrating a 22.7-fold inter-individual variation (0.65–14.78 N) that far exceeds material or pattern effects. Data are presented as mean ± standard error. Relative force denotes grip force normalized to body weight (%BW).

Analysis of six-axis force data revealed strong material–pattern interaction effects on grip force magnitude, but, critically, only under dynamic conditions. During dynamic movement, the force profile for each material showed a unique pattern-dependent shape: PLA peaked sharply at the Lizard pattern, PEBA peaked at the Pitcher pattern, while TPU maintained relatively stable force across all patterns (interaction *η*^2^ = 0.117) (Figure 5a). Under static conditions, this interaction weakened substantially (*η*^2^ = 0.037), with force profiles becoming more uniform across patterns within each material (Figure 5b). This biomechanical finding mirrors the perceptual weight reversal: dynamic conditions amplify material–pattern coupling, while static conditions decouple them. Individual grip force varied by 22.7-fold across participants (0.65–14.78 N), far exceeding the variation caused by material or pattern (Figure 5c). Representative biomechanical force time-series for both grip strategy types and force variability comparisons between conditions are shown in Supplementary Figure S2.

**Figure 6:**
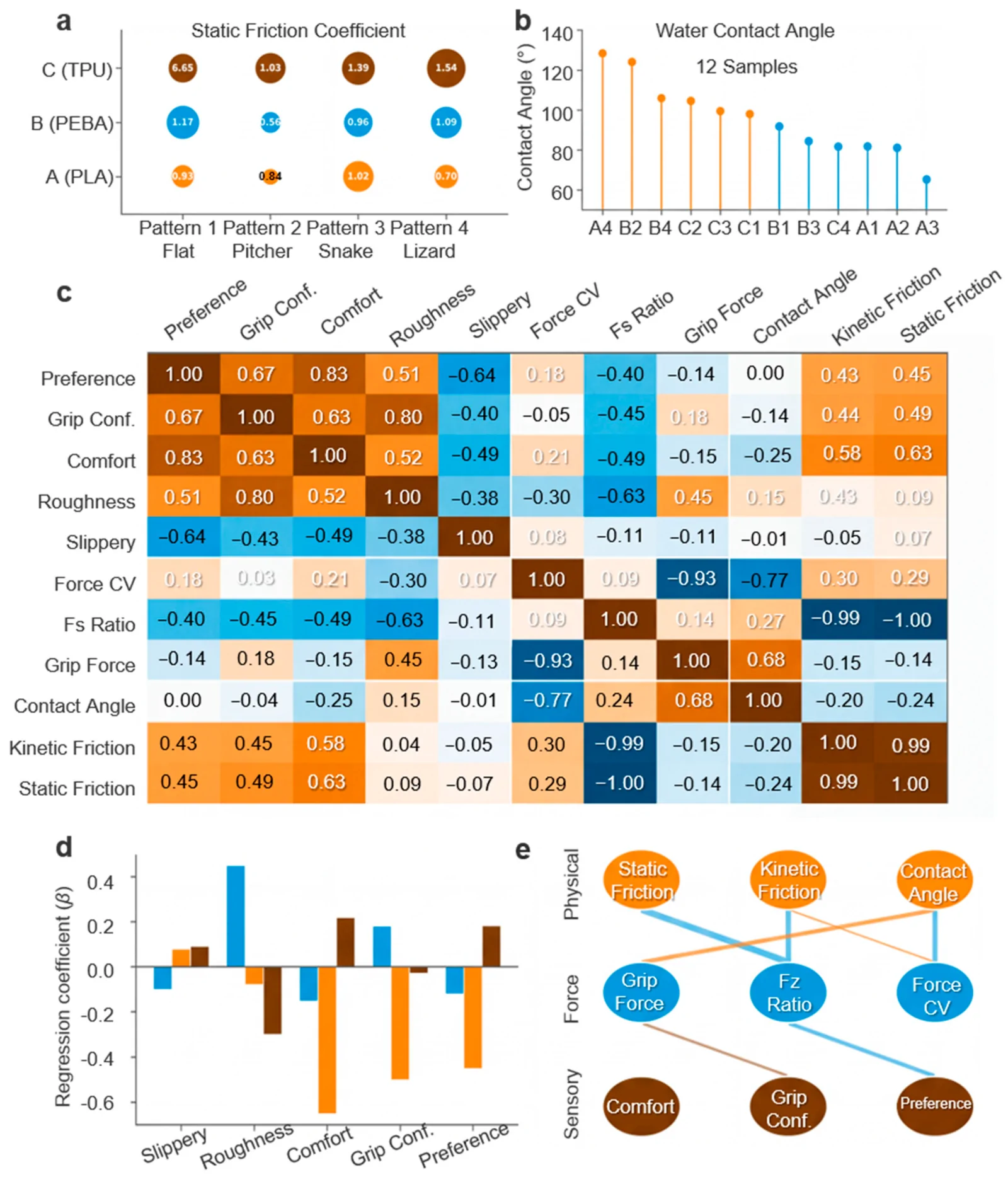
Physical characterization and multi-dimensional correlation structure. (**a**) Static friction coefficient matrix across 3 materials (A: PLA; B: PEBA; C: TPU) × 4 surface patterns (P1: Flat; P2: Pitcher; P3: Snake; P4: Lizard), visualized as a bubble matrix. Friction coefficients range from 0.42 (A2) to 1.54 (C4). (**b**) Water contact angle for 12 hold designs, presented as a lollipop plot sorted by magnitude (range: 65.3–128.3°). (**c**) 12 × 12 correlation heatmap integrating physical properties (friction, contact angle), biomechanical metrics (grip force, force variability), and sensory dimensions (slippery, roughness, comfort, confidence, preference), revealing a modular layered structure. (**d**) Force–sensory regression coefficients (β) from linear mixed-effects models across five sensory dimensions; the corresponding Pearson correlation coefficients between objective force metrics and subjective perception were near-zero (all |r| < 0.11). (**e**) Three-layer association network illustrating the association chain from physical properties through biomechanical force to sensory perception, with progressively decreasing mean correlation strength across layers (physical–force: |r| = 0.455; force–sensory: |r| = 0.254).

Static friction coefficients ranged widely from 0.42 to 1.54 across material–pattern combinations, with TPU + Lizard showing the highest friction and PLA + Pitcher the lowest. Water contact angle ranged from 65.3° to 128.3°. Friction and contact angle showed no significant correlation (R^2^ = 0.057, *p* = 0.453), indicating that these physical properties capture independent aspects of surface behavior. The full correlation matrix across 12 indicators from three domains—physical, biomechanical, and sensory—revealed a clear modular structure (Figure 6a–c). Force–sensory analysis found near-zero Pearson correlations between objective force metrics and subjective sensory ratings across all five dimensions (all |r| < 0.11); the corresponding regression coefficients (β) from linear mixed-effects models reached magnitudes up to approximately −0.65 and 0.45 (Figure 6d). This near-independence means that what climbers feel is not a direct readout of how much force they apply [36], suggesting substantial cognitive and perceptual processing between mechanics and experience. The three-layer association network quantified these associations: physical properties moderately correlated with biomechanical force patterns (mean |r| = 0.455), whereas force patterns only weakly correlated with sensory perception (mean |r| = 0.254) (Figure 6e). The decreasing correlation strength across layers implies partial independence between objective mechanics and subjective experience. Additional physical property correlation analyses, including static versus kinetic friction and friction–preference relationships, are provided in Supplementary Figure S3.

### 3.4. Inter-Individual Variability and Data-Driven Grip Strategy Classification

**Figure 7:**
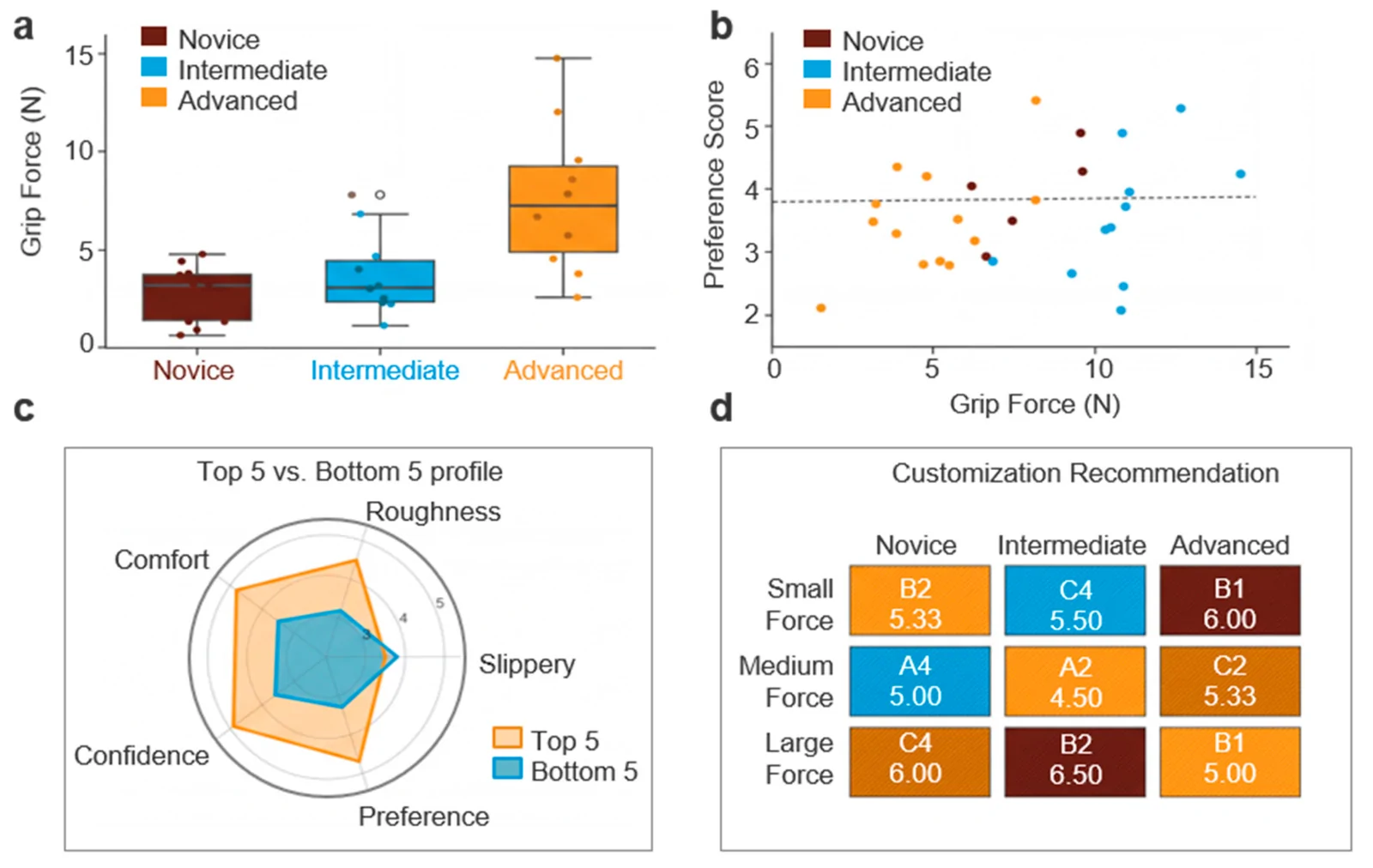
Individual differences and data-driven customization framework. (**a**) Grip force distribution across three skill levels (novice: gray; intermediate: blue; advanced: red), demonstrating a 22.7-fold inter-individual variation (0.65–14.78 N). (**b**) Scatter plot of grip force magnitude versus preference rating, showing near-zero correlation (r = 0.035) and confirming that force production and subjective preference are independent dimensions. Dashed lines indicate group means. (**c**) Radar chart comparing the five-dimensional sensory profiles of the top 5 (orange) and bottom 5 (blue) hold designs, revealing that preferred holds balance grip security with comfort rather than maximizing friction alone. (**d**) Customization recommendation matrix (3 × 3) mapping optimal hold designs across grip force magnitude levels (low, medium, high) and skill levels, with each cell indicating the best-performing hold and its preference score. This framework translates statistical patterns into actionable, personalized hold design guidance enabled by 3D printing technology.

Grip force magnitude varied by 22.7-fold across participants (0.65–14.78 N), representing the single largest source of variation. Advanced climbers tended to produce higher forces, but overlap between skill levels was substantial. Grip force magnitude showed virtually no correlation with preference rating (r = 0.035), confirming that force and preference are independent dimensions (Figure 7a). Comparing the sensory profiles of the top 5 and bottom 5 holds revealed that preferred holds balance grip security with comfort rather than maximizing friction alone (Figure 7b). The low dynamic–static preference consistency across individuals (mean r = 0.072) further underscores the complexity of individual preference. The four-tier grip force classification and individual participant rankings are detailed in Supplementary Table S7. Individual 12-hold preference heatmaps under dynamic and static conditions, along with within-participant preference consistency distributions, are presented in Supplementary Figure S4.

**Figure 8:**
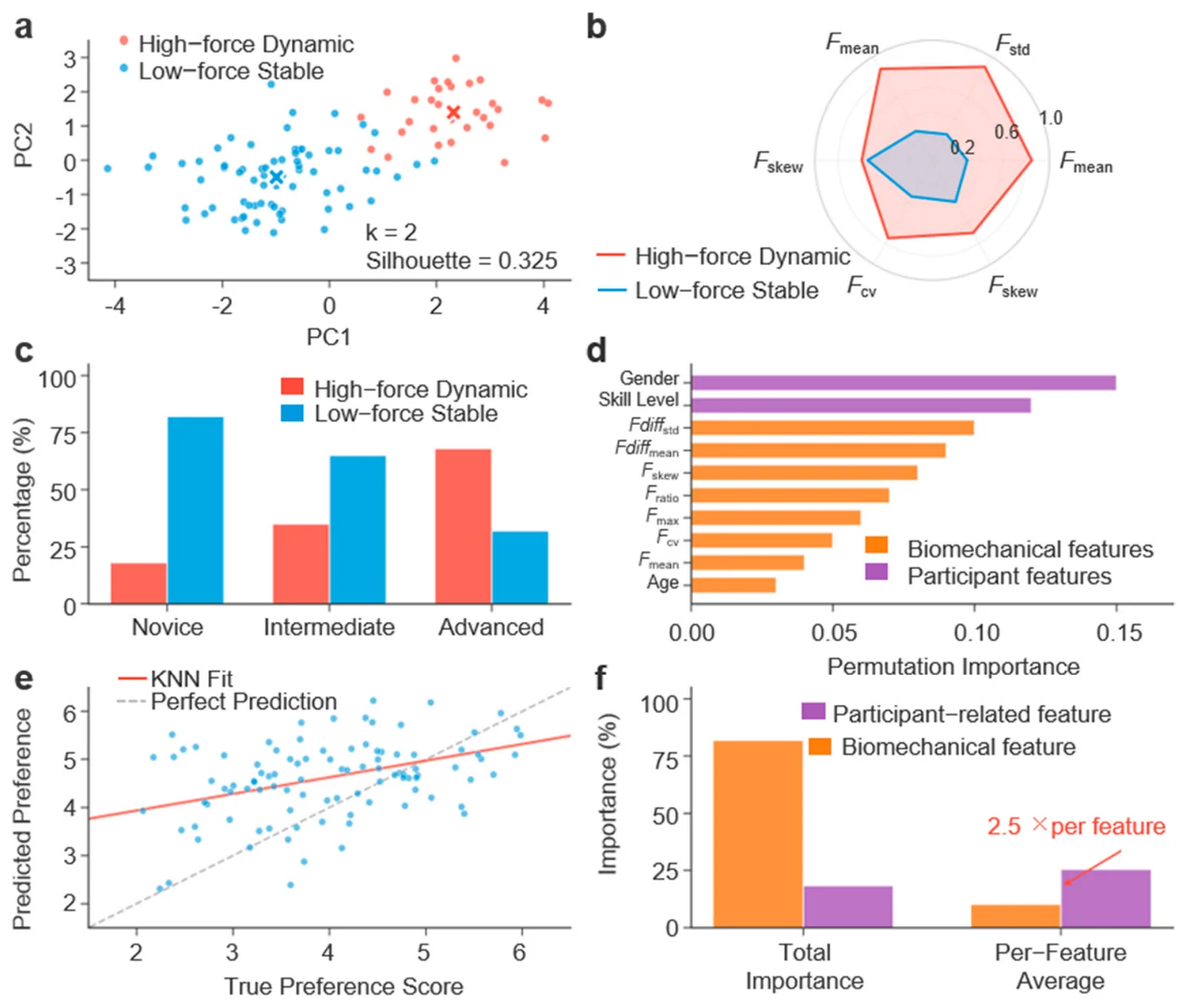
Machine learning analysis of grip strategies and preference prediction. (**a**) K-means clustering visualized via principal component analysis, identifying two distinct grip strategy types: High-force Dynamic (27.5%) and Low-force Stable (72.5%). (**b**) Comparison of thirteen biomechanical features between the two grip strategy types, showing divergent force application patterns. (**c**) Stacked bar chart showing the distribution of grip strategies across three skill levels, with advanced climbers predominantly adopting the High-force Dynamic strategy (68%). (**d**) Relative feature importance of biomechanical and participant-related features in the prediction model of sensory perception. Biomechanical features contributed 81.7% of total predictive importance. (**e**) K-nearest neighbors (KNN) model performance for preference prediction, achieving R^2^ = 0.190 and MAE = 1.139. (**f**) Comparison of per-feature mutual information between participant-related features (n = 32) and biomechanical features (n = 13). On average, each participant-related feature carries ~2.5× mutual information relative to each biomechanical feature.

K-means clustering revealed two distinct grip strategy types (Figure 8a): a High-force Dynamic Type (27.5% of trials) characterized by large, variable force production, and a Low-force Stable Type (72.5% of trials) marked by smaller, more consistent force application (silhouette coefficient = 0.325). This finding aligns with grasp synergy research in motor control [37,38], which has demonstrated that human grasping behavior can be decomposed into distinct motor primitives [39,40]. Strategy distribution varied dramatically across skill levels: 68% of advanced climbers’ trials used the high-force dynamic strategy, compared to only 18% for novices (Figure 8c). Feature importance analysis (Figure 8d) demonstrated that biomechanical features dominated the prediction of sensory perception, contributing 81.7% of the total predictive importance. In contrast, mutual information analysis (Figure 8f) evaluated information quantity on a per-feature basis. The average mutual information of each participant-related feature was approximately 2.5 times that of each biomechanical feature. These two analyses address distinct research questions: feature importance quantifies predictive contribution in the regression model, whereas mutual information describes intrinsic information content of individual features. KNN regression achieved moderate predictive performance (R^2^ = 0.190, MAE = 1.139) for preference rating, confirming that preference is partially predictable but also contains substantial idiosyncratic variation consistent with preference learning theory [41]. Detailed comparisons of biomechanical features between the two grip strategy types and their skill-level distributions are provided in Supplementary Table S8. Complete KNN feature importance rankings, including aggregate and per-feature comparisons, are listed in Supplementary Table S9.

## 4. Discussion

We formulated three hypotheses to address gaps in the literature on climbing holds: (H1) surface pattern will exert a stronger influence than material stiffness on climbers’ sensory perception during dynamic climbing, whereas material stiffness will become the dominant perceptual factor during static grasping, reflecting a dynamic–static perceptual weight reversal; (H2) material–pattern interaction effects on grip force will be stronger under dynamic than under static conditions, providing a biomechanical parallel to the perceptual weight reversal; and (H3) individual characteristics will modulate these effects, such that sex influences surface-pattern preference, skill level is associated with distinct grip-force strategies, and grip force magnitude is largely independent of sensory preference. To test these hypotheses, we integrated physical, biomechanical, and perceptual analyses of climbing holds under both static and dynamic conditions while explicitly accounting for individual differences. Testing 12 hold designs (3 materials × 4 surface patterns) on 30 climbers stratified across three skill levels, we addressed three interrelated gaps: the siloed treatment of physical, biomechanical, and perceptual layers; the dominance of static testing in a fundamentally dynamic activity; and the persistent reliance on group averages. Three principal findings emerged: a dynamic–static perceptual weight reversal; a three-layer association model linking physical, biomechanical, and sensory layers; and systematic individual differences that exceed material or pattern effects. Together, these findings inform a data-driven customization framework for 3D-printed climbing holds.

Several limitations should be noted. The sample was drawn from a single population, restricting demographic breadth. The 12 hold designs and the laboratory protocol limit generalizability to outdoor and competition settings. The static and dynamic tasks also differed in duration, muscle contraction mode, and fatigue level (a 10 s passive hang versus repeated concentric pull-ups); although task order was counterbalanced and the order effect was not statistically significant, these task-level confounds cannot be fully separated from the movement-state effect, so the static–dynamic differences should be interpreted with appropriate caution. The cross-sectional design cannot capture how grip strategy evolves with training, age, or injury history. These constraints frame the interpretation of the results that follows.

The dynamic–static perceptual weight reversal is, to our knowledge, the first empirical evidence that the relative perceptual weight of material versus surface pattern shifts with movement state in climbing. Because the dominant effect sizes were drawn from different sensory dimensions across states (the pattern effect on roughness during dynamic movement and the material effect on comfort during static grasping), we interpret this finding as a state-dependent reweighting of perceptual factors rather than a strict within-dimension crossover. The most parsimonious interpretation is that dynamic and static contexts engage distinct information-processing pathways: when the hand is in motion, cutaneous cues from surface microtexture dominate because the climber cannot rely on prolonged contact, whereas static grasping allows time for material stiffness to be perceived through deeper proprioceptive channels. This finding extends prior work showing that climbers judge hold surfaces by perceived grippiness rather than roughness, with grippiness—but not roughness—correlating with friction coefficients. Whereas that study established a perceptual-physical decoupling, the present results add a state-dependent axis: the same material that feels optimal in a static hang may be perceptually dominated by surface texture during dynamic movement. The reversal is also consistent with coordination research showing that skill differences are most pronounced during dynamic sequences. The practical implication is that route setters and equipment designers should specify the intended movement state when selecting holds, since a hold chosen for static recovery may underperform when used dynamically.

The three-layer association model (physical → biomechanical → sensory) provides a descriptive account of why subjective feel does not always track objective friction. The progressively decreasing correlation strength across layers (mean |r| = 0.455 for physical–force and |r| = 0.254 for force–sensory) indicates that each interface introduces noise and individual variation, dampening the signal from material to experience; these cross-sectional correlations describe associations and cannot establish causal direction. This observation resonates with tribological work on climbing shoe rubber, which emphasized the complexity of surface interactions, and with broader critiques of reductionist paradigms in motor behavior. The present results extend these observations by quantifying, for the first time in a climbing-specific context, the magnitude of correlation loss at each interface. The partial coupling between biomechanical force and sensory rating also explains an observation familiar to practitioners but rarely examined empirically: that high-friction materials can feel “sticky” rather than “secure,” and low-friction materials can feel “clean” rather than “slippery.” The model therefore serves as both a diagnostic tool (identifying which layer is the bottleneck for a given climber) and a design principle (optimizing across all three layers rather than the surface alone).

Inter-individual variation in grip force (a 22.7-fold range) far exceeded the effects of material or pattern, addressing the persistent neglect of individual differences in climbing equipment research. This result echoes evidence that climbers vary greatly in strength, anthropometry, and perceptual sensitivity, and that sex differences in climbing physiology are well documented. The identification of two grip strategy types—High-force Dynamic and Low-force Stable—extends grasp synergy analysis to climbing-specific contexts and shows that inter-individual variation takes systematic, classifiable forms. The variation in strategy type with skill level, combined with sex-related differences in pattern preference, indicates that grip behavior is shaped by physiological capacity, training history, and perceptual style. Average-based design heuristics, which have dominated hold development, are therefore unlikely to satisfy more than a minority of users.

The integration of these three findings into a data-driven customization framework represents a methodological shift from population-level optimization to individual-level matching. The framework leverages the customization capability of 3D printing and aligns with the increasing use of machine learning in sports biomechanics for pattern recognition and grasp analysis. Unlike conventional hold selection, which prescribes fixed material–pattern combinations, the proposed framework prescribes a design process: (i) profile the climber (force capacity, strategy type, perceptual preference); (ii) profile the usage context (static vs. dynamic); and (iii) match hold design to the resulting profile. This process-aware approach has direct applications in training prescription, injury rehabilitation, and ergonomic equipment design in adjacent sports.

In summary, this study shows that (i) the perceptual weight of material versus surface pattern reverses with movement state, (ii) physical, biomechanical, and sensory layers form a partially coupled association chain in which signal strength decreases at each interface, and (iii) inter-individual variation in grip behavior is both large and systematic. These findings call for a shift from population-based to climber-specific hold design and provide the empirical and conceptual basis for a process-aware customization framework enabled by 3D printing.

## Figures and Tables

**Table 1:** Participant demographic characteristics by sex (mean ± SD).

Characteristic	Total (n = 30)	Male (n = 19)	Female (n = 11)
Age (years)	23.6 ± 3.4	23.6 ± 3.9	23.5 ± 2.3
Height (cm)	172.3 ± 7.6	176.9 ± 5.0	164.5 ± 3.9
Body mass (kg)	63.8 ± 9.8	69.7 ± 6.9	53.5 ± 3.8
Climbing experience (years)	6.0 ± 6.8	6.3 ± 7.3	5.6 ± 6.3

**Table 2:** Participant demographic characteristics by skill level (mean ± SD).

Characteristic	Novice (V1–V3) (n = 10)	Intermediate (V4–V6) (n = 10)	Advanced (V7–V10) (n = 10)
Age (years)	22.3 ± 3.6	22.7 ± 2.1	25.7 ± 3.4
Height (cm)	173.2 ± 6.8	171.9 ± 10.5	171.9 ± 5.3
Body mass (kg)	65.0 ± 11.4	61.8 ± 9.8	64.5 ± 8.9
Climbing experience (years)	1.9 ± 3.9	3.8 ± 2.4	12.5 ± 7.6

**Table 3:** Dynamic Sensory Evaluation Ratings by Skill Level (Mean ± SD).

Group	Slippery	Roughness	Comfort	Grip Confidence	Preference
Novice (n = 10)	3.68 ± 1.40	4.11 ± 1.40	4.06 ± 1.27	4.20 ± 1.39	3.92 ± 1.43
Intermediate (n = 10)	3.77 ± 1.35	3.80 ± 1.38	4.08 ± 1.59	4.19 ± 1.64	3.89 ± 1.60
Advanced (n = 10)	3.66 ± 1.34	3.70 ± 1.32	4.52 ± 1.30	4.65 ± 1.12	4.10 ± 1.48

## Data Availability

The data that support the findings of this study are available from the corresponding author upon reasonable request.

## Supplementary Materials

The following supporting information can be downloaded at: https://www.mdpi.com/article/10.3390/s26216863/s1, Table S1: Shore hardness and tensile modulus of the three filament materials; Table S2: FDM 3D printing parameters for three filament materials; Table S3: Six-axis force/torque sensor specifications; Table S4: Sensory evaluation dimensions and verbal anchors (9-point Likert scale); Table S5: Gender-specific pattern preference scores (dynamic condition); Table S6: Post-hoc pairwise comparisons (Tukey HSD adjusted) from the linear mixed-effects models; Table S7: Individual grip force distribution: four-tier classification; Table S8: K-means clustering: two grip strategy types; Table S9: KNN feature importance for preference prediction; Figure S1: Group-level sensory evaluation comparison (3 skill levels × 2 conditions); Figure S2: Biomechanical force time-series examples; Figure S3: Physical property correlation analysis; Figure S4: 12-hold preference ranking under dynamic and static conditions (individual participant data). Reference [42] is cited in the Supplementary Materials.
