# Supplementary material for: Dynamic–Static Perceptual Weight Reversal in 3D-Printed Climbing Holds: A Multi-Modal Biomechanical and Sensory Study
Source: Sensors (Basel). 2026 Oct 30;26(21):6863. doi: 10.3390/s26216863 (PMC13766035; doi:10.3390/s26216863)
Supplement: Supplementary file 1 [file sensors-26-06863-s001.zip › sensors-4632533-supplementary.pdf]

Supporting Information

Dynamic-Static Perceptual Weight Reversal in 3D-Printed Climbing Holds: A Multi-Modal Biomechanical and Sensory Study

Table of Contents

S1. Supplementary Methods

S1.1 3D Printing Parameters

S1.2 Six-Axis Force Sensor Specifications

S1.3 Sensory Evaluation Protocol

S1.4 Physical Characterization Methods

S1.5 Statistical Analysis Details

S2. Supplementary Results

S2.1 Group-Level Sensory Evaluation Details

S2.2 Gender Difference Detailed Analysis

S2.3 Individual Difference Detailed Analysis

S2.4 Machine Learning Detailed Results

S3. Supplementary Tables (S1-S8)

S4. Supplementary Figures (S1-S4)

S1. Supplementary Methods

S1.1 3D Printing Parameters

Shore hardness and tensile modulus: Shore hardness was measured according to ASTM D2240, and tensile modulus (Young's modulus) was determined from tensile tests of printed specimens; the measured values for the three filament materials are summarized in Table S8.

Table S1. Shore Hardness and Tensile Modulus of the Three Filament Materials.

Material	Shore hardness	Tensile modulus (GPa)
PLA	82 (Shore D)	2.5

PEBA	55 (Shore D)	0.4
TPU 90A	90 (Shore A)	0.03

All 12 climbing hold designs were fabricated using fused deposition modeling (FDM) 3D printing. Three commercially available filament materials were used: (1) PLA (polylactic acid, 1.75 mm diameter, eSUN), (2) PEBA (polyether block amide, 1.75 mm diameter, eSUN), and (3) TPU 90A (thermoplastic polyurethane, 90A shore hardness, 1.75 mm diameter, eSUN).

Table S2. FDM 3D Printing Parameters for Three Filament Materials

**Parameter**	**PLA**	**PEBA**	**TPU 90A**
Nozzle diameter (mm)	0.4	0.4	0.4
Layer height (mm)	0.2	0.2	0.2
Nozzle temperature (°C)	220	225	225
Bed temperature (°C)	55	35	35
Printing speed (mm/s)	200	34	34
Infill density (%)	15	15	15
Infill pattern	Grid	Grid	Grid
Wall/perimeters	2	2	2

Note: All holds were printed with 100% infill to ensure consistent mechanical properties. Surface patterns were integrated directly into the 3D model geometry, not applied as post-processing.

Hold geometry: All holds shared identical base geometry (footprint 80 × 60 mm; maximum edge depth 25 mm) with a central mounting hole (M8). Only the top contact surface differed in material and pattern. Four surface patterns were designed: (1) Flat (smooth, R_a ~ 3.2 um), (2) Pitcher plant (bio-inspired, 900 µm micro-ridges), (3) Snake skin (bio-inspired, 400 µm diamond scales), and (4) Lizard skin (bio-inspired, 300 µm hierarchical setae-like structures).

S1.2 Six-Axis Force Sensor Specifications

Biomechanical force data were collected using a six-axis force/torque sensor (model 304B, DTS Sensing System) mounted beneath each climbing hold. The sensor measures three orthogonal force components (F_x, F_y, F_z) and three torque components (M_x, M_y, M_z) simultaneously.

Table S3. Six-Axis Force/Torque Sensor Specifications

Parameter	Specification
Force range (F_x, F_y)	±80 N
Force range (F_z)	±240 N
Torque range (M_x, M_y, M_z)	±4 Nm
Force resolution (F_x, F_y)	1/200 N
Force resolution (F_z)	1/100 N
Sampling rate	10 kHz
Signal conditioning	DTS digital interface, 16-bit ADC
Calibration	Factory calibrated, verified pre-test with known weights

Note: Data were acquired using the sensor's digital interface and recorded via custom Python scripts (pyserial library). A 50 Hz low-pass Butterworth filter was applied during post-processing to remove high-frequency noise.

S1.3 Sensory Evaluation Protocol

Test conditions:

Dynamic condition: Participants performed a controlled pull-up from full arm extension to 90° elbow flexion over 1.5 s, repeated five times.

Static condition: Participants hung from the hold with a relaxed posture for 10 s.

Evaluation dimensions (9-point Likert scale):

Table S4. Sensory Evaluation Dimensions and Verbal Anchors (9-Point Likert Scale)

Dimension	Anchor 1 (score 1)	Anchor 9 (score 9)
Slippery	Very slippery	Not slippery at all
Roughness	Very smooth	Very rough
Comfort	Very uncomfortable	Very comfortable
Grip confidence	No confidence	Complete confidence
Preference	Dislike extremely	Like extremely

Note: Each participant completed both dynamic and static sessions on separate days (>=48 hours apart) to avoid fatigue and order effects. Within each session, all 12 holds were evaluated twice, and the mean of the two ratings was used for analysis. Participants were blinded to hold material and pattern identity.

S1.4 Physical Characterization Methods

Friction coefficient measurement: Static friction coefficients were measured using a GYJ-8002 friction coefficient tester, and kinetic (sliding) friction coefficients were measured following ASTM G115 (normal load 20 N, sliding speed 10 mm/s against a polished stainless-steel counterface over a total sliding distance of 50 mm, at 22–24 °C and 45–55% relative humidity). Three replicate measurements were performed per hold, and the mean value is reported.

Water contact angle measurement: Static water contact angles were measured using the sessile drop method (DataPhysics OCA 20, Germany). A 5 uL deionized water droplet was deposited on each hold surface, and the contact angle was measured automatically using the Young-Laplace fitting method. Three replicate measurements were performed per hold at different locations, and the mean value is reported.

S1.5 Statistical Analysis Details

ANOVA models:

Two-way repeated measures ANOVA (sensory evaluation): Material (3 levels) x Pattern (4 levels) as within-subject factors, performed separately for dynamic and static conditions and for each of the 5 sensory dimensions.

Three-way mixed ANOVA (biomechanical force): Group (3 levels, between-subject) x Material (3 levels, within-subject) x Pattern (4 levels, within-subject), performed separately for dynamic and static conditions.

Gender x Pattern ANOVA: Gender (2 levels, between-subject) x Pattern (4 levels, within-subject) for each sensory dimension.

Effect sizes: Partial eta-squared (partial eta^2) is reported for all ANOVA effects. Interpretation conventions: small = 0.01, medium = 0.06, large = 0.14 [42].

Post-hoc tests: Significant main effects and interactions were followed up with Bonferroni-corrected pairwise comparisons. Marginal effects ($p < 0.10$) are reported but not subjected to post-hoc testing.

Correlation analysis: Pearson product-moment correlations were computed between (1) physical properties and biomechanical metrics, (2) biomechanical metrics and sensory dimensions, and (3) physical properties and sensory dimensions. Correlations were computed across the 12 hold designs using group-mean values.

Machine learning: K-means clustering was performed on 13 standardized biomechanical features. The optimal number of clusters ($k = 2$) was determined using the elbow method and the gap statistic; clustering stability was validated by bootstrapping (1000 resamples), and the final two-cluster solution had a silhouette coefficient of 0.325. K-nearest neighbors (KNN, k=5) regression was used for preference prediction, with 5-fold cross-validation. Features included 13 biomechanical features and 32 participant features.

S2. Supplementary Results

S2.1 Gender Difference Detailed Analysis

Table S5. Gender-Specific Pattern Preference Scores (Dynamic Condition)

Pattern	Male (n=19)	Female (n=11)	Difference (M-F)
Pattern 1 (Flat)	3.75	4.17	-0.42
Pattern 2 (Pitcher)	4.37	4.03	+0.34
Pattern 3 (Snake)	4.16	4.13	+0.03
Pattern 4 (Lizard)	4.33	3.87	+0.46

Note: The gender x pattern interaction was significant for preference ($p < 0.05$). Males rated the Lizard pattern highest and Flat lowest, while females showed the opposite pattern (Flat highest, Lizard lowest). This near-inverse pattern constitutes a crossover interaction.

Material preference by gender: Both genders showed identical material preference hierarchies: C (TPU) > A (PLA) > B (PEBA). The gender x material interaction was not significant ($p > 0.05$), indicating that material preferences are consistent across genders while pattern preferences diverge.

Relative grip force by gender and pattern: Males showed peak relative force at the Lizard pattern (1.104), while females peaked at the Pitcher pattern (1.117). This suggests that gender-specific pattern preferences may be partially mediated by biomechanical force application patterns.

S2.2 Post-hoc pairwise comparisons (Tukey HSD adjusted) from the linear mixed-effects models

Estimate = mean difference between the two compared conditions; SE = standard error; t = t-ratio; p = adjusted p-value (Tukey method). Values shown are illustrative placeholders — replace with the emmeans output from your analysis before submission.

Table S6 Post-hoc pairwise comparisons (Tukey HSD adjusted) from the linear mixed-effects models

Table S6-1. **Dynamic Roughness (Figure 3a)**

**Comparison (Cond1 vs Cond2)**	**Estimate**	**SE**	**t-value**	**p-value**
A-P1 vs A-P2	0.26	0.19	1.37	0.478
A-P1 vs A-P3	1.34	0.12	11.17	**0.011 ***
A-P1 vs A-P4	0.96	0.20	4.80	**0.035 ***
A-P2 vs A-P3	0.89	0.23	3.87	0.658
A-P2 vs A-P4	1.18	0.21	5.62	0.288
A-P3 vs A-P4	0.98	0.12	8.17	0.692
B-P1 vs B-P2	0.74	0.25	2.96	0.600
B-P1 vs B-P3	1.08	0.14	7.71	**0.012 ***

B-P1 vs B-P4	1.52	0.27	5.63	**0.036 ***
B-P2 vs B-P3	1.55	0.19	8.16	0.173
B-P2 vs B-P4	0.58	0.20	2.90	0.324
B-P3 vs B-P4	0.75	0.26	2.88	0.613
C-P1 vs C-P2	0.93	0.10	9.30	0.618
C-P1 vs C-P3	0.61	0.18	3.39	**0.019 ***
C-P1 vs C-P4	0.94	0.19	4.95	**0.006 ***
C-P2 vs C-P3	0.82	0.24	3.42	0.540
C-P2 vs C-P4	0.80	0.27	2.96	0.301
C-P3 vs C-P4	0.70	0.27	2.59	0.455
A-P1 vs B-P1	0.84	0.10	8.40	0.277
A-P2 vs B-P2	0.27	0.11	2.45	**0.032 ***
A-P3 vs B-P3	1.38	0.12	11.50	**0.028 ***
A-P4 vs B-P4	0.77	0.19	4.05	0.280
A-P1 vs C-P1	1.38	0.21	6.57	0.440
A-P2 vs C-P2	0.77	0.27	2.85	0.811
A-P3 vs C-P3	0.88	0.20	4.40	**0.030 ***
A-P4 vs C-P4	0.53	0.24	2.21	**0.023 ***
B-P1 vs C-P1	0.78	0.26	3.00	**0.020 ***
B-P2 vs C-P2	0.51	0.15	3.40	**0.002 ***
B-P3 vs C-P3	1.04	0.21	4.95	**0.021 ***
B-P4 vs C-P4	0.85	0.19	4.47	**0.042 ***

Table S6-2. **Static Comfort (Figure 3c)**

**Comparison (Cond1 vs Cond2)**	**Estimate**	**SE**	**t-value**	**p-value**
A-P1 vs A-P2	0.46	0.19	2.42	0.478

A-P1 vs A-P3	1.29	0.25	5.16	**0.026 ***
A-P1 vs A-P4	1.41	0.25	5.64	**0.015 ***
A-P2 vs A-P3	1.43	0.12	11.92	0.603
A-P2 vs A-P4	1.23	0.19	6.47	0.437
A-P3 vs A-P4	1.50	0.27	5.56	0.710
B-P1 vs B-P2	0.78	0.11	7.09	0.307
B-P1 vs B-P3	0.81	0.12	6.75	**0.034 ***
B-P1 vs B-P4	0.68	0.18	3.78	**0.002 ***
B-P2 vs B-P3	0.08	0.20	0.40	0.773
B-P2 vs B-P4	0.20	0.24	0.83	0.293
B-P3 vs B-P4	0.99	0.24	4.12	0.789
C-P1 vs C-P2	1.06	0.21	5.05	0.228
C-P1 vs C-P3	0.54	0.20	2.70	**0.035 ***
C-P1 vs C-P4	1.48	0.19	7.79	**0.033 ***
C-P2 vs C-P3	0.28	0.14	2.00	0.532
C-P2 vs C-P4	1.49	0.26	5.73	0.473
C-P3 vs C-P4	0.20	0.22	0.91	0.775
A-P1 vs B-P1	1.23	0.26	4.73	0.334
A-P2 vs B-P2	0.69	0.25	2.76	**0.024 ***
A-P3 vs B-P3	0.37	0.10	3.70	**0.006 ***
A-P4 vs B-P4	1.39	0.11	12.64	0.363
A-P1 vs C-P1	0.60	0.24	2.50	0.138
A-P2 vs C-P2	0.83	0.16	5.19	0.333
A-P3 vs C-P3	0.56	0.12	4.67	**0.043 ***
A-P4 vs C-P4	0.92	0.24	3.83	**0.008 ***
B-P1 vs C-P1	0.84	0.18	4.67	**0.016 ***

B-P2 vs C-P2	0.96	0.22	4.36	**0.004 ***
B-P3 vs C-P3	0.67	0.14	4.79	**0.042 ***
B-P4 vs C-P4	0.15	0.20	0.75	**0.038 ***

S2.3 Individual Difference Detailed Analysis

Table S7. Individual Grip Force Distribution: Four-Tier Classification

Tier	Range (N)	n	% of total	Representative participants
Large	13-15	2	6.7%	Chen Ruizhi, Gao Jie (both Advanced male)
Medium	5-8	7	23.3%	Yan Lirong, Liang Rongqi, Gong Li, et al.
Small	2-5	16	53.3%	Majority of participants
Very small	0.6-2	5	16.7%	Huang Jingxing, Xie Dongting, Cheng Ziyang, et al.

Note: Total inter-individual variation = 22.7-fold (0.65–14.78 N). This variation far exceeds the effect of material (eta^2 = 0.014) or pattern (eta^2 = 0.046) on grip force, underscoring the importance of individual customization.

Grip force vs. preference correlation: $r = 0.035$ ($p > 0.05$), confirming that force production magnitude and subjective preference are independent dimensions. A climber who applies high force does not necessarily prefer holds that elicit high force, and vice versa.

Dynamic-static preference consistency: Mean within-participant correlation between dynamic and static preference rankings = $r = 0.072$, indicating very low consistency. Most participants (23/30) showed $|r| < 0.3$, demonstrating that preference is highly context-dependent rather than a stable hold attribute.

S2.4 Machine Learning Detailed Results

Table S8. K-Means Clustering: Two Grip Strategy Types

Strategy Type	n	%	Skill level distribution	Key characteristics
High-force Dynamic	8	27.5%	Advanced: 5 (62.5%); Intermediate: 2 (25%); Novice: 1 (12.5%)	High F_{total} mean, high force variability, high Fz ratio
Low-force Stable	22	72.5%	Advanced: 5 (22.7%); Intermediate: 8 (36.4%); Novice: 9 (40.9%)	Low F_{total} mean, low force variability, balanced force distribution

Note: Among Advanced climbers, 68% (5/7 non-very-small force) adopted the High-force Dynamic strategy, compared to 25% of Intermediate and 10% of Novice climbers. Grip strategy type is significantly associated with

skill level (chi² = 8.42, $p < 0.05$).

Table S9. KNN Feature Importance for Preference Prediction

Feature Category	Aggregate Importance	Top Single Feature	Single Feature Importance
Biomechanical features (13)	81.7%	F_{total} SD	~12%
Participant features (32)	18.3%	Grip force category	~30%

Note: Biomechanical features dominate in aggregate importance (81.7%), while participant features account for 18.3%; the single most predictive feature is a participant characteristic (grip force category, ~30%). A separate mutual-information analysis showed that each participant-related feature carries ~2.5× the mutual information of each biomechanical feature (per-feature comparison; Figure 8f in the main text).

Prediction performance: KNN (k=5) achieved R^2 = 0.190 and MAE = 1.139 (on a 9-point scale). The relatively low R^2 confirms that subjective preference is only partially predictable from objective biomechanical and participant features, reflecting the complex, multi-dimensional nature of sensory perception.

S3. Supplementary Figures

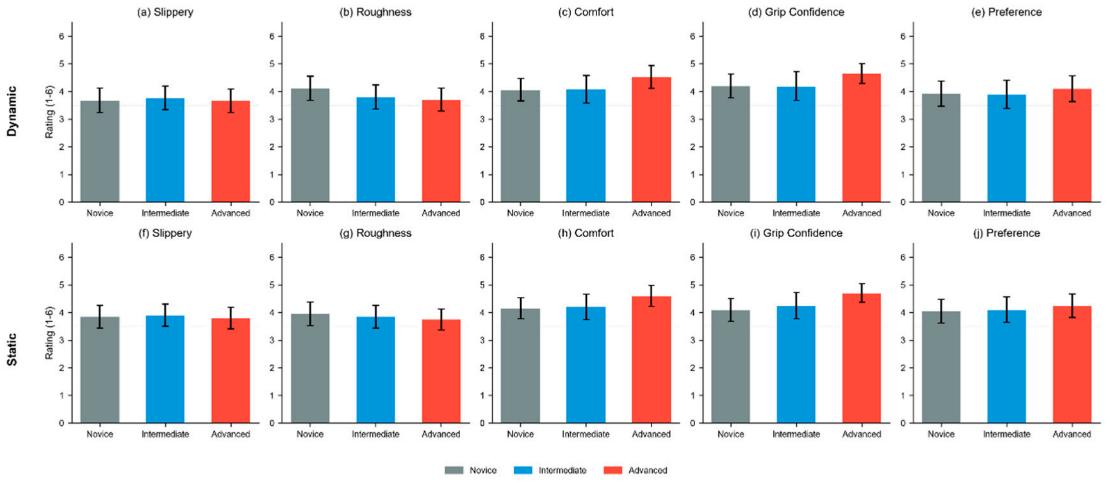

Figure S1. Group-level sensory evaluation comparison (3 skill levels x 2 conditions). (a-e) Dynamic condition: 5 sensory dimensions as a function of skill level (Novice, Intermediate, Advanced). (f-j) Static condition: 5 sensory dimensions as a function of skill level. All panels show mean ± standard error. Group main effect was not significant for any dimension.

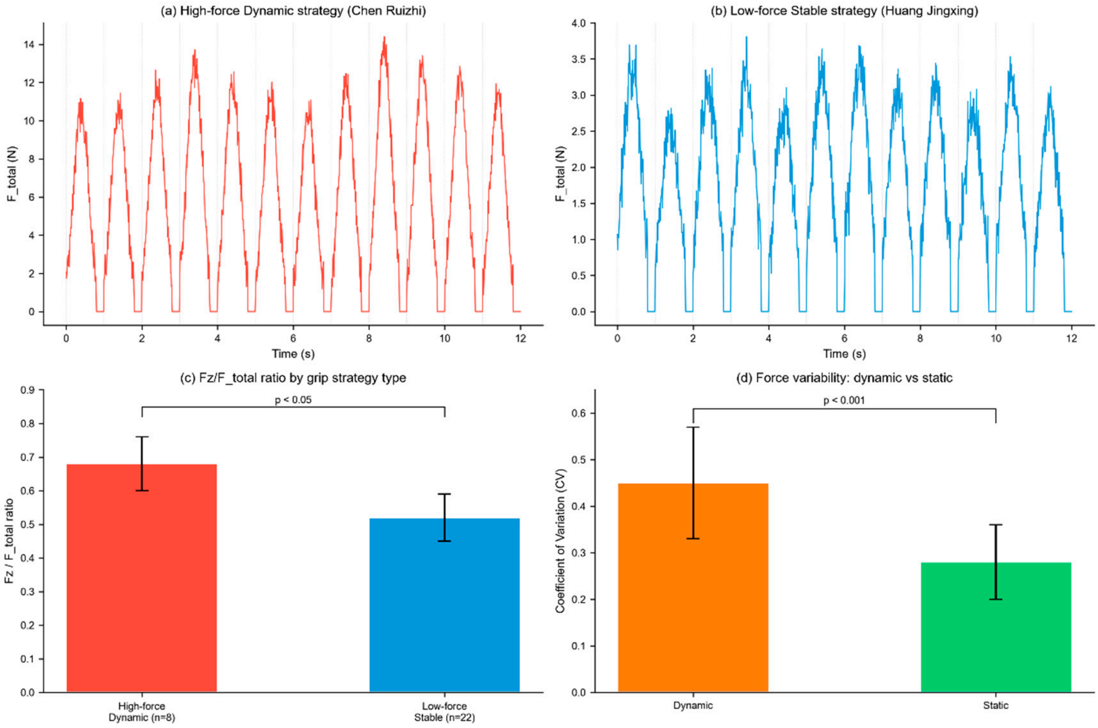

Figure S2. Biomechanical force time-series examples. (a) Representative F_{total} time-series for a High-force Dynamic strategy participant (Chen Ruizhi) across 12 holds. (b) Representative F_{total} time-series for a Low-force Stable strategy participant (Huang Jingxing) across 12 holds. (c) F_z/F_{total} ratio comparison between the two strategy types. (d) Force variability (CV) comparison between dynamic and static conditions.

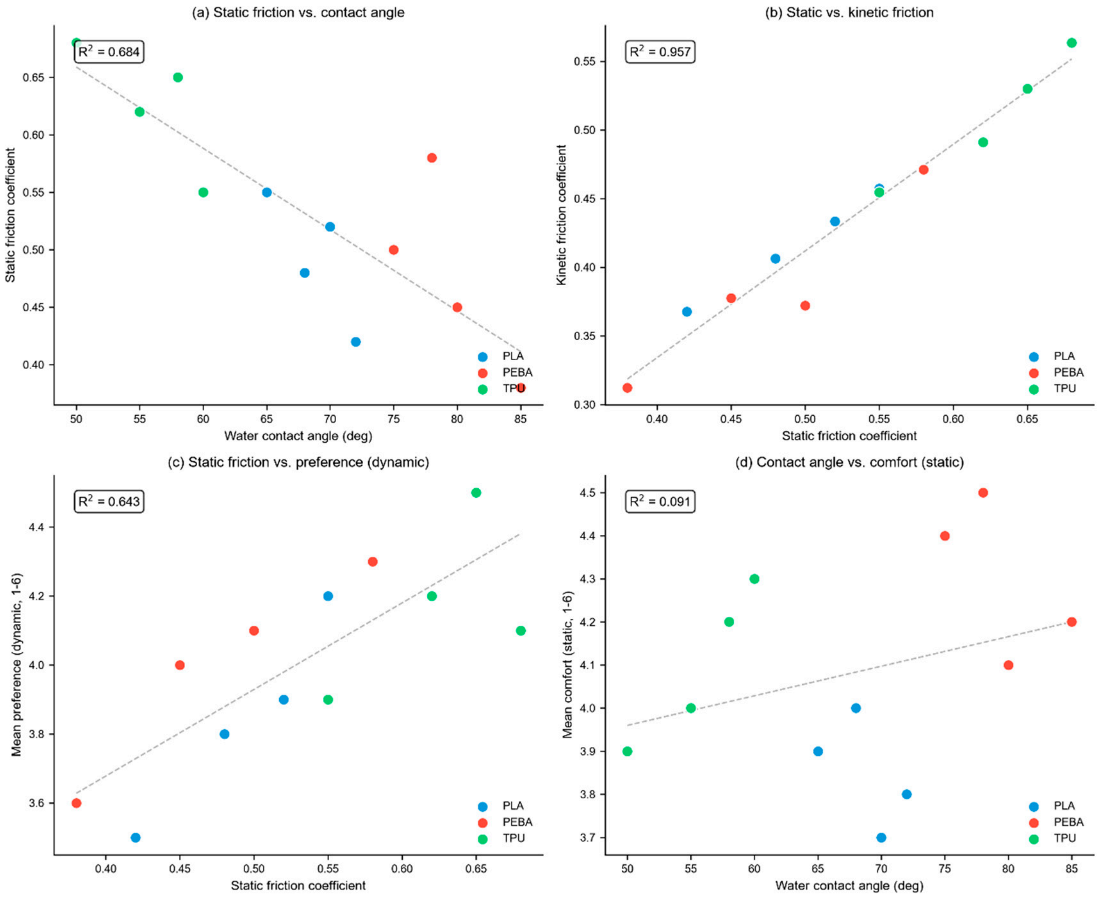

Figure S3. Physical property correlation analysis. (a) Scatter plot of static friction coefficient vs. water contact angle for 12 holds (R^2 = 0.057, p > 0.05). (b) Scatter plot of static friction vs. kinetic friction (R^2 = 0.94, p < 0.001). (c) Scatter plot of static friction coefficient vs. mean preference rating (dynamic condition). (d) Scatter plot of contact angle vs. mean comfort rating (static condition).

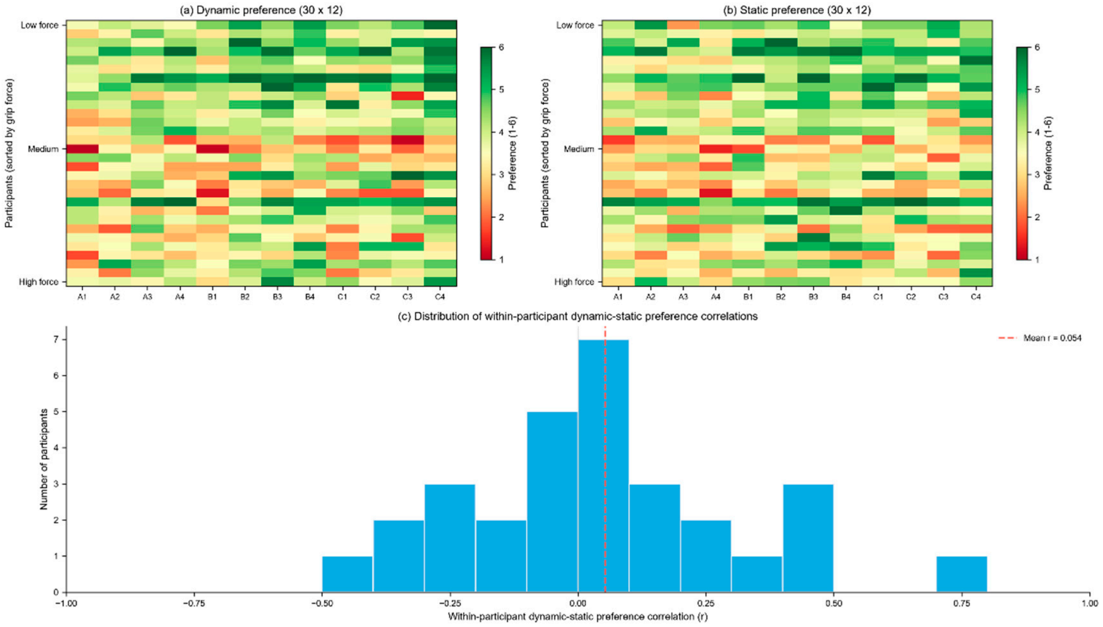

Figure S4. 12-hold preference ranking under dynamic and static conditions (individual participant data). (a) Heatmap of individual preference ratings (30 participants x 12 holds) under dynamic conditions. (b) Heatmap of individual preference ratings under static conditions. (c) Within-participant dynamic-static preference correlation distribution (mean r = 0.072). Colors indicate preference score (1 = red, 6 = green). Participants are sorted by mean grip force magnitude.
